# Commercial Surrogacy: An Overview

**DOI:** 10.1055/s-0042-1759774

**Published:** 2022-12-29

**Authors:** Pedro Brandão, Nicolás Garrido

**Affiliations:** 1 Department of Reproductive Medicine, Instituto Valenciano de Infertilidad, Valencia, Spain; 2 University of Valencia, Valencia, Spain; 3 IVI Foundation, Valencia, Spain

**Keywords:** bioethics, fertilization in vitro, legislation medical, medical tourism, surrogacy, bioética, fertilização em vitro, legislação médica, turismo médico, gestação de substituição

## Abstract

**Objective** Surrogacy is the process in which a woman carries and delivers a baby to other person or couple, known as intended parents. When carriers are paid for surrogacy, this is known as commercial surrogacy. The objective of the present work is to review the legal, ethical, social, and cultural aspects of commercial surrogacy, as well as the current panorama worldwide.

**Methods** This is a review of the literature published in the 21 ^st^ century on commercial surrogacy.

**Results** A total of 248 articles were included as the core of the present review. The demand for surrogate treatments by women without uterus or with important uterine disorders, single men and same-sex male couples is constantly increasing worldwide. This reproductive treatment has important ethical dilemmas. In addition, legislation defers widely worldwide and is in constant change. Therefore, patients look more and more for treatments abroad, which can lead to important legal problems between countries with different laws. Commercial surrogacy is practiced in several countries, in most of which there is no specific legislation. Some countries have taken restrictive measures against this technique because of reports of exploitation of carriers.

**Conclusion** Commercial surrogacy is a common practice, despite important ethical and legal dilemmas. As a consequence of diverse national legislations, patients frequently resort to international commercial surrogacy programs. As of today, there is no standard international legal context, and this practice remains largely unregulated.

## Resumo

**Objetivo** A gestação de substituição é o processo no qual uma mulher engravida e entrega um bebê a outra pessoa ou casal, conhecidos como pais pretendidos. Quando as gestantes são pagas, isto é conhecido como gestação de substituição comercial. O objetivo do presente trabalho é rever os aspectos legais, éticos, sociais e culturais da gestação de substituição comercial, bem como o panorama atual em todo o mundo.

**Métodos** Trata-se de uma revisão da literatura publicada no século XXI sobre a gestação de substituição comercial.

**Resultados** Um total de 248 artigos foi incluído nesta revisão. A demanda por tratamentos com gestação de substituição por mulheres sem útero ou com distúrbios uterinos importantes, homens solteiros e casais masculinos está aumentando constantemente em todo o mundo. Este tratamento reprodutivo tem dilemas éticos importantes. Além disso, a legislação é amplamente adiada em todo o mundo e está em constante mudança. Portanto, os pacientes procuram cada vez mais por tratamentos no exterior, o que pode levar a importantes problemas legais entre países com leis diferentes. A gestação de substituição comercial é praticada em vários países, na maioria dos quais não há legislação específica. Alguns países tomaram medidas restritivas contra esta técnica por causa de relatos de exploração destas mulheres.

**Conclusão** A gestação de substituição comercial é uma prática comum, apesar de importantes dilemas éticos e legais. Como consequência de diversas legislações nacionais, os pacientes frequentemente recorrem a programas de gestação de substituição comercial internacionais. Atualmente, não existe um contexto jurídico internacional padrão e esta prática permanece em grande parte não regulamentada.

## Introduction

Surrogacy is the process in which a woman carries and delivers a baby to another person or couple, known as the commissioning or intended parents (IPs). 1 2 The first historical report of surrogacy is in the book of Genesis – the case of Sarah and Abraham's child, Ishmael, carried by their servant. 3 However, the first officially recognized surrogacy was performed in 1991.

Traditional or genetic surrogacy occurs when the carrier also provides the oocyte, hence she is the genetic female progenitor of the child. This modality has progressively been abandoned and the American Society of Reproductive Medicine (ASRM) clearly recommends against it. 4 On the other hand, in gestational surrogacy, also called treatment with a gestational carrier (GC), both gametes are provided by other people, so the surrogate woman has no genetic links with the child. 5 Nomenclature regarding this matter may be somewhat misleading – some also call this last modality “partial” (and traditional would be total surrogacy) since the carrier is only providing the womb, while others call it “total” because the embryo is completely genetically unrelated to the carrier (and traditional would be partial surrogacy). 6 Other terms have been used less frequently, such as classical or straight. 7

Gestational carriers may be a relative, a friend, or a person chosen by the surrogacy agency or in vitro fertilization (IVF) clinic. In the last case, the surrogacy process may be completely anonymous or there may be a direct contact between the IPs and the surrogate. 8 Intended parents may be heterosexual couples, female couples or single women, usually with a uterine condition that limits pregnancy, as well as male couples or single men.

Surrogacy may be altruistic or gestational carriers may be paid for the process, which is called “commercial” or “compensated” surrogacy.

Commercial surrogacy has been practiced for the last decades and is usually associated with important costs. In general lines, there is a written agreement which outlines both the intentions of both parties, defines duties, delineates reimbursements and payments, and allows decision-making when contingencies occur. 9

There is neither an absolute number of babies born through his technique, nor an exact estimation of its value. In the beginning of the decade of 2010, an estimated 2% of all the IVF cycles in the United States of America (USA) and Canada ended in an embryo transfer to a gestational carrier, 18% of them by foreign patients. In California, in 2015, the percentage of foreign patients was estimated to be 44%. 10 11 12 In the early 2000s (it was legal by then), the commercial surrogacy business was evaluated by Thai and Indian national departments of health as 125 and 449 million US dollars (USD), respectively. 13 In 2012, the industry of surrogacy in the USA was estimated to be worth around 6 billion USD. 5 There was a 4-fold increase in the number of GC cycles in the USA between 1999 and 2013. Between 2006 and 2010, there was a 1,000% increase in the market of international surrogacy. There are reports of international surrogacy agencies stating a growth of 6,000% in 12 years. 14

In the USA, a complete process of surrogacy may cost as much as $200,000. Programs usually include $20,000–80,000 for medical expenses, $3,000–15,000 for legal support, $6,000–54,000 for surrogacy recruiting programs and $20,000–55,000 for carrier compensation. 15 In low-income countries, a full surrogacy process usually costs less than half of the USA. 16

The aim of the present work is to review and summarize information published in scientific journals about commercial surrogacy, with particular attention to the legal, ethical, and sociocultural aspects of this reproductive treatment as well as current practices worldwide.

## Methods

This is a review of all articles listed in PubMed concerning commercial surrogacy published in the 21 ^st^ century. The search was conducted in November 2021 using the query “surrogacy” and limiting to articles published from 2000 (including) on. Articles not related to commercial surrogacy were excluded, as well as editorials, letters to the editor, comments, corrigenda, replies, book chapters and study protocols. Articles written in English, French, Portuguese, or Spanish were included. References of the selected articles were thoroughly reviewed in order to include other potentially related articles.

### Study Appraisal

From the search using the query, a total of 1,278 results were retrieved. All titles and/or abstracts of the articles were analyzed. Duplicates were removed ( *n =* 3). Studies not related to the study question ( *n* = 965), editorials/letters to the editor ( *n =* 7) and answers or comments ( *n =* 17) were excluded. From the remaining articles, 5 mentioned only altruistic surrogacy, 28 were not available (mainly because of the year or journal of publication) and 5 were excluded due to language. At end, a total of 248 articles were included as core of this review.

## Ethical Issues

### Principle of Autonomy

If a surrogate treatment is performed by free will of both intended parents and carrier, one may assume that autonomy is guaranteed. 17 If surrogacy was to be prohibited, both IPs and surrogate would be restrained from having the option to participate in it, which violates their autonomy and free will. 18 Nevertheless, the offspring is not autonomous to take any decision on the matter, and will be restricted the right to completely know his/her biological origins. 19

### Principle of Beneficence

In the same way, if intended parents wish to have a child, their benefit is obvious. The gestational carrier may have her benefit from her personal satisfaction of aiding others and considerable economic compensation. The offspring will be granted the right to live. 20

### Principle of Nonmaleficence

On the other hand, IPs will face important costs, they won't be able to experience pregnancy, they will not have any control on the surrogate or pregnancy, and they will be imposed a potential additional stress caused by the distance to their children-to-be. Couples seldom report fears related to the surrogate process, particularly related to social judgment and legal troubles arising from this procedure. 21 However, studies on IPs show they usually have a good experience throughout the surrogacy process. 22 Carriers will be exposed to the risks of pregnancy, may eventually suffer from social stigma and ostracism (including by their family, which often leads them to move away from their communities to hide pregnancy) and will not have any motherhood rights on that child. 23 24 25 26 27 While studies with surrogates in high-income countries show that GCs perceive surrogacy as a positive experience, studies in low-income countries show high rates of depression and negative feelings in GCs. 28 29 30 A curious comparison has been made between the carrier and a nanny – if the child is already adopted prenatally by the IPs, the surrogate would play the role of a "prenatal nanny”. These authors question the potential maleficence of being a carrier and ask if it would be more outrageous to be a nanny before birth than after. 31 Children may eventually suffer from social stigma and may have difficulties when being told their true origins. They will not be able to know or have any contact with their birth mother. 32 33 Nevertheless, studies about the opinion of children (born after surrogacy) are reassuring, even though evidence to date is limited. 24 34 35 In addition, some doubts have been raised about raising a child in a non-traditional (non “mother and father”) family. 36 However, much research has been done on child rearing by same-sex couples and the results are reassuring. 37

### Principle of Justice

If all people were to have the same access to surrogacy, the principle of justice would be granted for everyone, but this implies that surrogacy is not to be limited based on marital status, ethnicity, religion, sexual orientation, or any another kind of discrimination. 38 39 In fact, surrogacy is a way to provide fertility to some infertile couples, singles and same-sex couples. 40 However, in case of commercial surrogacy, the costs are high and not affordable by everyone. As opposed to patients, carriers are not given any legal motherhood rights. In addition, the process of selecting only healthy young women as carriers may lead to discrimination of other candidates willing to participate. 41 As opposed to conventionally-born children, children born through surrogacy are not granted the right to grow with their gestational mother. 17

### Ethical Aspects of Commercial Surrogacy

An important ethical aspect of commercial surrogacy is that women may be regarded as a “way to conception” and children as mere products of conception. 17 42 43 44 45 Concerning children, some regard surrogacy as “selling babies” or human trafficking. 46 47 48 49 Others consider it does not violate any of the children's rights, it cannot be regarded as a market of babies and that if the conditions of the surrogate arrangement are fulfilled at the end of the process, the best interest of the child is implicitly protected, since this was the manifested desired of both parties, a carrier who was always aware she was not going to be mother, and the IPs who are receiving their most desired child. 50 Some consider these treatments to be exploitative to women. 51 52 53 In fact, commercial surrogacy opens a door to illegal exploitation if not adequately ruled and monitored, especially in low-income countries. 54 55 In many cases, third party organizations or people receive their compensation and little, if any, is given to the surrogate. 56 57 58 59 It is also not uncommon that women are not aware of the risks of this procedure and do not have an opinion on the decision to become a carrier. 60 61 62 Others regard surrogacy as a different way of prostitution. 63 64 Surrogacy is also seldom compared to donating or selling a kidney *in vivo* . 25 65 66 Interestingly, some argue that paid surrogacy is in no worse position than many other exploitative commercial transactions which take place against a backdrop of global inequality and constrained options, such as poorly paid and dangerous construction work. Hence, there would be no reason for special condemnation. 67 The criminalization of commercial surrogacy may result in undesirable consequences, removing opportunity for evidence-based law reforms which would regulate the process. 68 69 70 71 In the end, some authors argue that the theoretical “do no harm” reasons to refuse surrogacy are far from being proven. Thus, there would be no reasons for banning commercial surrogacy. 72 73 74

### Motivations

There are several reasons that may lead women to become a GC, such as economic compensation, pure altruism, the wish of going through a different kind of motherhood or cultural beliefs. 63 75 Nevertheless, the majority of carriers undergo this process for the compensation, mostly people with dependent families. 76 77 78 In fact, some women tend to accept it as a work and regard the surrogacy agency as their “boss”. 23 76 79 Other gestational carriers perceive this process as an “exchange of gifts”. 79 On the other side, patients who resort to surrogacy are usually women (single or part of a couple) without uterus or with important uterine disorders impairing pregnancy, single men, and same-sex male couples. 80 81 82 It is not uncommon that clinics, agencies or international intermediates advertise commercial surrogacy treatments specifically to single men and male couples. 83

### Compensation

Depending on national legislation, surrogacy may be commercial or altruistic. Nevertheless, in some countries where only altruistic surrogacy is allowed, carriers may be given a compensation for specific matters related to the process (such as health care expenses, sick leaves, etc.). However, given the considerable costs of healthcare in some countries, a large reward could eventually be acceptable as a mere compensation for these expenses. Thus, it may not be easy to clearly define the border between altruistic and commercial. Depending on the maximum amount allowed (if any), these compensations are seldom used off the record to mask monetary payment for surrogacy. 84 As a consequence, many authors consider the distinction between ‘altruistic’ and ‘commercial’ surrogacy increasingly unsustainable both in law and policy. 85 The amounts given to carriers in a commercial surrogacy process vary widely between countries. In the USA a gestational carrier usually receives an average amount of USD 20,000 to 55,000 per pregnancy. 15 86 Multiple pregnancies are usually paid a supplement. A monetary compensation may be regarded as a win-win situation for both parties, as the surrogate gets the money and the IPs get the child, while some believe surrogacy must not be reduced to a business transaction. 87 88 The monetary compensation for surrogacy may lead to a contradiction. On the one hand, paying a low amount may be regarded as compensation for expenses and damage, but also as an exploitation. 89 90 On the other hand, paying higher amounts leads carriers to be better compensated for their efforts, but may also lead to a competitive reproductive market, “machinerizing” of women and treating children and reproductive treatments as commodities. 51 In the Netherlands, some attempts have been made to define lower and upper limits for compensations, mainly based on the oocyte donation models. However, it is not easy to define what would be the true labor associated with surrogacy and if it should be considered as a full or partial-time job, since women will be pregnant 24 hours a day, but they are able to combine surrogacy with their daily activities, including other jobs. 51 Especially in low-income countries, it is not uncommon for surrogates to expect an extra-contractual compensation for the process. 87 In 2005, the European Society of Human Reproduction and Embryology (ESHRE) published its position regarding commercial surrogacy – “Payment for services is unacceptable; only reimbursement of reasonable expenses and compensation for loss of actual income should be considered”. 91 The International Federation of Gynecology and Obstetrics (FIGO) also stated that surrogate arrangements should not be commercial. 92 93 On the other hand, the American Society of Reproductive Medicine (ASRM) compares gestational surrogacy to medical research, in which individuals are paid for activities demanding time, stress, physical effort and risk, so they consider financial compensation for surrogacy ethically justifiable. 4 Likewise, the American College of Obstetricians and Gynecologists (ACOG) affirms that compensation is ethical and appropriate for the time, effort and risks taken by a gestational carrier. 94

### Anonimity Regimen

In many states, anonymity of surrogacy is to be guaranteed, which means the choice of the surrogate mother and all the communication between her and the IPs is indirect and mediated by the clinic or agency. This is the perfect regimen for some couples who prefer anonymity and not to know the carrier in any point of the process. 95 On the other hand, there are some states where carriers and IPs are not only obliged to know and approve each other, after checking they match their expectations, but they are also encourage to actively communicate during the process and participate during all its steps. 87 96 Some authors believe this involvement between IPs and carriers and an eventual further relationship of the latter with the child may be beneficial to all parties and may ease some of its ethical issues. 97 98

### Medical Risks

Being a gestational carrier is associated with important adverse medical or psychological outcomes. Obstetric complications are not higher (if not lower) in surrogate singleton gestations, since surrogate mothers are usually young and healthy. 99 100 101 102 103 Nevertheless, no gestation is exempt from risk. 104 105 Also, double embryo transfers are quite common in surrogate processes, because it is usually cheaper than having two separate pregnancies, resulting in more multiple pregnancies. 106 107 108 109 In addition, it is believed that cesarean section rates are high among surrogates, not only because IPs may ask for it, but also because low-income surrogates receive medical care in private clinics while in other situation they would be treated in public health systems. 110 Candidates to |GC are often quite misinformed about the procedure and lack of psychological and legal support. 111 Particularly in developing countries, women are seduced into being GCs. Many of these women live in precarious conditions and use this resource for a better future for themselves and their families. In some cases, women are even forced to be GCs. 112 In some countries, such as India, gestational carriers often live in a hotel hired for the purpose, in order to have more dignified conditions, a healthier lifestyle for their pregnancy and more easily be able to maintain obstetric surveillance, particularly women who live in remote areas. 113 Likewise, children born from surrogacy are not risk free. Nevertheless, current scientific data suggest this option is safe as long as all parties have adequate screening and medical, psychological, and social supports. 99 114 In order to optimize the outcomes of a surrogate gestation, both the United States Food and Drug Administration (FDA) and the ASRM have developed guidelines to help choosing the most adequate gestational carrier. 115 Ideally, candidates to gestational carriers must be between 21 and 45 years old, with an optimal BMI, have at least one previous term uncomplicated pregnancy, but no more than 5 deliveries or 3 cesarean sections and with a 12 to 18 months pregnancy interval. The optimal selection of GC candidates also includes assessment of their mental health, since this may be a very demanding process. 116 Adequate medical counseling to the surrogate candidates must be done in order to promote healthy habits both before and during pregnancy. Women must be encouraged to receive preconception immunizations, if applicable, to avoid potential teratogenic medications, to take folic acid supplements, to restrain from smoking, drinking alcohol, and excessive caffeine intake. 92

### Surrogacy Agencies and Marketing

There are several international agencies exclusively dedicated to intermediate surrogate treatments. 117 The websites of these agencies seldom advertise surrogacy treatments abroad focusing on the needs of IPs, referring to surrogacy as a solution to their problem, privileging genetic parenthood. Many online advertisements of global medical tourism offer "special deals" on commercial surrogacy. 118 119 They seldom include basic and guarantee plans. The difference is that the latter includes all necessary embryo transfer to have a live newborn. The potential for exploitation of the carriers is obviously not exposed and the surrogacy arrangements are advertised as a mutual benefit. In fact, this subject is often a taboo and avoided as much as possible during all the surrogacy process. 120 Surrogacy agencies usually include staff trained in international legislation and marketing. Interestingly, most of the staff of these agencies have also undergone a similar process or is quite familiar with other transnational reproductive treatments by personal experience. 121 They usually provide legal assistance, included in all their plans. Regardless of the countries and their legal context, it is not uncommon for these agencies to advertise that there are no legal risks and there will be no litigation. They take it for granted that the surrogates have no legal rights over the child-to-be, that both the country of treatment and the country of origin will only recognize the motherhood of IPs. These agencies also state that in case of litigation, the law always protects the IPs, when actually in most cases there is no legal framework. 122 However, these agencies are an important means for IPs to easily reach a surrogacy contract, including recruitment of donors, carriers, reproductive treatments, obstetric follow-up, and legal assistance. 123 124

### Legal Issues

Legal conflicts may appear in the country where surrogacy is performed, but also in the country of origin of the IPs (“receiving country”), when returning home with the child. 125 126 127

### Country Where Surrogacy is Performed

National legislation varies substantially worldwide. 128 Some countries explicitly prohibit any type of surrogacy, others allow surrogacy of any type, while others have some restrictions concerning marital status, sexual orientation, nationality, country of residence, medical reason to undergo a surrogate treatment and the altruistic/commercial nature of the process. In most countries, surrogacy is not regulated at all. 14 All surrogacy arrangements beginning by signing a contract between the IPs and the GC. There are innumerous important points that should be clearly settled in the contract in order to avoid future potential litigation. 129 These include setting out both parties legal parentage and nonparentage rights, agreements on prenatal and delivery issues, compensations and fees, insurances, and assumptions of risks. 130 The central and most important party in any reproductive treatment is the offspring because he/she is the only party that cannot have a word in any preconception contract or agreement. As a consequence, most countries worldwide recognize that the child, regardless of the way in which he/she was conceived, has the same rights guaranteed by the national and international framework of human rights. 131 132 Regarding the mother, defining biological motherhood may be quite challenging in the modern era, especially in assisted reproductive treatment (ART) involving third parties, such as donated gametes or surrogacy. An interesting example is the reception of oocytes from partner (ROPA) method, or lesbian shared IVF, in which both women share biological motherhood, one will be the gestational mother (the one giving birth) and the other will be the genetic mother (the one providing the oocytes). 133 In surrogacy, 3 people may be involved in motherhood: the carrier (which will be the birth mother), the oocyte provider and the intended mother (depending if it is with own or donated oocytes, these last two will be the same or different women, respectively). 134 In the majority of countries, legal motherhood is based upon the fact of birth. The “anonymous” or “secret birth,” where a woman may choose to give birth without revealing her identity, is not legal in most countries. Thus, as a rule, the woman giving birth is automatically recognized as mother, until proven otherwise. The requirement for a man to be registered as a father of a child depends upon the circumstances of the case, especially the couple's marital status. In most countries, in a married heterosexual couple, the man is automatically assumed as the father. However, in most cases, a man may voluntarily acknowledge his legal paternity. Once a child is registered and receives a birth certificate, parents are legally recognized as so for all purposes. However, in most states, it is possible to reverse this process upon genetic proof. 135 One of the main obstacles for couples who resort to surrogacy is the registration of the newborn in their name and the cession of motherhood rights by the carrier. 136 Countries where surrogacy is contemplated by law, as is the case of some states of the USA, a prepregnancy contract is signed between the two parties in which the surrogate waives any rights to motherhood after birth. Therefore, in these cases, the birth certificate is automatically conceived with the name of the intended parents. On the other hand, in countries where surrogacy is not regulated and it is performed not because it is legal, but because it is not illegal, the original birth certificate is usually issued with the surrogate as mother, and the IPs have to ask national authorities to amend the certificate with their names. 137 However, litigation may arise in various points throughout the surrogacy journey, in view of obstetric complications, decisions regarding pregnancy interruption, lack of agreement between the IPs and the surrogate, divorce or separation of the IPs, or changes of mind of one of the parties during the process. 138 Even in the presence of a prior contract, if this practice is not regulated and there is no specific legislation, its legal value is doubtful. The most troublesome scenario is if the surrogate decides not to abdicate her motherhood rights. 139 140 141 In these cases, a DNA test is seldom required. Thus, parenthood is determined on a genetic basis and the court is asked to declare the motherhood rights of the carrier null. In some cases, parents have to wait months after birth to have the birth certificate amended. 142 This may be even more problematic if pregnancy is a result of double donation (donated oocytes and sperm), in which none of the IPs shares a genetic link with the baby. In any case, in the absence of a deferment by a court, the carrier has full motherhood rights over the child, which prevents the child from leaving the country with the IPs without her consent. There are reports of large amounts of bribes paid to the carrier to finally cooperate by ceding her rights. 143

### Receiving Country

Due to the absence of uniform international legislation, cross-border surrogacy treatments may pose legal issues when returning to the home country of IPs with children who, according to the legislation of the receiving country, have been conceived illegally. 144 145 146 147 The main steps where IPs face difficulties most frequently in their home countries are when requiring a passport or any travel documentation at their consular authorities overseas to return home with the child, and when the IPs, back home, wish to register their children as a national citizen. 148 If their native countries do not recognize surrogacy, patients may struggle to register the child as theirs. 149 150 151 152 Further problems may arise in cases of singles or same-sex couples from countries where they are not allowed to have children. In these cases, surrogacy itself may not be the sole problem, but the lack of legal framework to recognize both same-sex IPs as legal parents. 143 There are reports of people who were criminally accused of having filed an illegal process abroad. Nevertheless, national courts ended up acquitting them for lack of legal support regarding international affairs, because these procedures were officially recognized in the country they were performed, and because this decision was ultimately considered to be in the best interest of all parties involved, especially the child. 143 153 154 As a consequence of these disparities between legislations and issues of countries regarding international private laws, many judicial authorities of several states have attempted to create solutions to enable children born from an international surrogacy arrangement to return home. The Hague Conference on Private International Law (HCCW) is an intergovernmental organization in the area of private international law that administers international conventions, protocols, and legal instruments. It is an important organization that deals with conflicting international affairs. In 2012, the Permanent Bureau of the HCCW released “A Preliminary Report on The Issues Arising from International Surrogacy Arrangements”. Since then, this institution has been trying to create guidelines to standardize the international recognition of surrogacy performed abroad. As of 2021, the HCCW had 90 country as members. 14

### Transnational Surrogacy

The denial of surrogacy in most countries, for all or for some (such as single people or same-sex couples), its cost or the lack of available carriers led to an important transnational search for these (and other) reproductive treatments. 155 156 This phenomena has been called reproductive, procreative or fertility tourism, transnational reproduction or cross border reproductive care. 157 158 159 160 161 162 In European countries alone and concerning any kind of ART, in 2010, a total of 24,000 to 30,000 cycles of cross border fertility treatment within the continent were estimated each year, involving 11,000 to 14,000 patients. 163 Transnational surrogacy is one of the fastest-growing cross-border reproductive treatments. 164 Choosing where to perform the surrogacy treatment usually entails finding the right equilibrium between legal guarantees and costs. 165 Due to the variety of legislations, costs and availability of donors and carriers between countries, patients may search for other countries to do the entire process of surrogacy, or different phases of the surrogate treatment in more than one country. 158 As an example, a male couple may get their donated oocytes from South Africa, where there are many donors available, do the IVF, recruit the surrogate and embryo transfer in Georgia ( *Sakartvelo* ), due to attractive prices, and fly the gestational carrier to the USA to deliver the baby, where children may be registered by both parents. 166 167 Countries for gamete donation (when needed) are usually chosen based on the availability of donors, anonymity regimen of donation, costs of the process, compensation to the donors, and ethnic issues. In vitro fertilization, in turn, may pose some legal obstacles in some countries. Legal requirements, as well as costs, the possibility of freezing embryos, performing preimplantation genetic test (PGT) and sex selection are important aspects. Surrogacy itself is the most complex part of the process. The legal status of surrogacy is by far the most important aspect when it comes to choose the country, not only the presence or not of specific legislation concerning the matter, but also the legal value of surrogate contracts in more delicate situations, such as pregnancy interruption and in case the carrier decides to keep the baby. In addition, same-sex couples may choose the country of delivery in order to be able to share parenthood since birth. The exclusion of motherhood rights from the gestational carrier and the attribution of these rights to IPs may be done immediately after birth, or it may be a court decision after DNA tests to the child, genetic IPs and the carrier. 168 Furthermore, in the case of gay couples, the process of sharing legal parenthood may be much easier if their country of origin accepts joint adoption of a child by same-sex couples. It is very common to cross borders between neighboring countries to undergo surrogacy. Both parents or carrier may be required to cross the border, as well as gametes or embryos, depending on the case. Examples of frequent neighboring border crossings are between the USA and Mexico, and Thailand and Vietnam or Laos. 169 Diverse measures have been taken by many governments to avoid the so called “reproductive tourism”. Some countries where treatments used to be performed banned these treatments, at least for foreign patients. Other countries, such as Portugal, decided to approve surrogacy only to national or resident citizens since its very beginning, to avoid reproductive tourism and legal litigation with other countries. 170 On the other hand, receiving countries face important dilemmas when it comes to attribute nationality to the offspring, but they are also in an ungrateful position to limit reproductive treatments abroad. 171 The vast majority of countries have no specific legislation concerning children conceived abroad via surrogacy. 157 Some countries, such as Australia, the Netherlands or the UK, are trying to draw preconception agreements for surrogate treatments abroad. 84 Several scandals have been reported during the last decades, such as the Baby Gammy, a child with Down Syndrome, whose intended parents left him in Thailand while taking home his twin sister, who was not affected by the condition. 172 Another famous case was a Japanese man who tried to conceive seventeen children via surrogacy. 169 In India, a Japanese couple refused to receive the baby because they divorced 1 month before delivery. 173 Following these occurrences, some popular destinations, especially in Asia, have taken legal measures to limit commercial surrogacy or access to foreign patients. Commercial surrogacy was banned in Thailand and Nepal in 2015 and in Cambodia in 2016. 76 In India, same-sex couples were excluded in 2013 and in 2018 this country limited surrogacy to national patients. 166 174 Consequently, the offer of surrogacy destinations has decreased. Over time, the “one-stop” surrogacy destinations have become increasingly rare, especially due to the partial limitation of some of the steps of the process, requiring intended parents to do a “puzzle” with various countries to complete their journey in surrogacy. On the other hand, demand for surrogacy from high-income countries such as European countries and Australia is continuously rising, due to increasing maternal age, single men, and male same-sex couples.

### Cultural Aspects

Some social and cultural aspects influence the way society is more or less receptive to gestational surrogacy, especially the country of origin, religion, activism, and the whole social context. 175 176 Religion is one of the most important points, since different religions have various points of view regarding motherhood, marriage, life, and the status of the embryo. 177 Studies show that the vast majority of Muslims are against surrogate treatments, since procreation and childbearing must be carried out only under the framework of marriage. 178 179 However, some acknowledge this may be ethically justified and medically necessary. 180 Polls in Iran, Jordan, and Lebanon revealed a predominant negative attitude among healthcare workers and students toward surrogacy, mainly driven by religious beliefs. 181 182 183 In Jewish society, cases of donor eggs or surrogacy are also hard to deal with. If one of the women involved is not Jewish, rabbinic authorities disagree about the Jewish status of the child, which may imply that the child undergoes religious conversion. 184 The Catholic church is against any form of ART, especially if there is a third party involved, since reproduction is to be practiced in a marital context. Other branches of Christianism do accept IVF treatments. The opinions concerning surrogacy within Christians are diverse, even though they are, in general, in disfavor of this technique. 185 Hindus regard infertility as a curse, which means they accept ART and surrogacy as a cure for infertility. 185 Regarding Buddhism, since there are few theories written about ART, as long as pain and harm are avoided, all practices are acceptable. However, the very desire for a child through extraordinary means can also be seen as an unhealthy material attachment. As so, the matter of surrogacy is conflicting. 185 Studies report a duality of criteria in high income countries regarding public opinion about surrogacy. Poll-based studies in Australia, France, Germany, Japan, Philippines, Spain and the USA revealed that more than half of general population would be in favor of surrogate treatments for heterosexual and same-sex couples. 186 187 188 189 190 191 The same goes for reproductive care professionals and students. 192 193 194 On the other hand, feminists are against any kind of surrogacy. 63 195 196 Curiously, a recent meta-analysis showed that the majority of infertile women were not in favor of surrogacy. 197 198 A study in Romania revealed that women (general population) would rather adopt than resort to surrogacy. 199 On the other hand, studies with Iranian infertile couples reported that the majority has a positive view on surrogacy. 200 201

## Legal Context Worldwide

### America

**Fig. 1:**
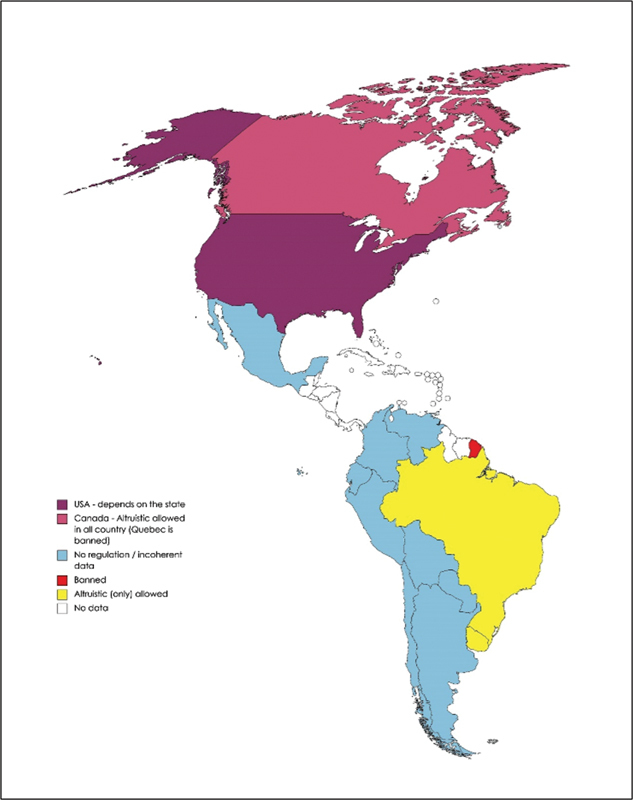
Map showing the current legal status of surrogacy in America.

By 2021, 22 USA states have no legal statutes for commercial surrogacy, 16 states explicitly and 7 states implicitly allow it, and in 5 states it is forbidden. 114 202 In Canada, commercial surrogacy is banned, even though altruistic surrogacy is permitted in all states except in Quebec. 5 203 204 In Mexico, legal status of surrogacy is not regulated at a federal level, thus, only a few states, like Tabasco, used to offer commercial arrangements. Consequently, Tabasco used to be a major destination for transnational surrogacy. In 2016, Tabasco changed the state law to limit surrogacy to heterosexual infertile couples. In June 2021, a Supreme Court decision upheld surrogacy in Mexico – the court endorsed both free and paid surrogacy and even invalidated the provisions of one state that prohibited access to same-sex and foreign couples. Since then, a door opened to any Mexican state to perform commercial surrogacy agreements. 205 In most countries of South America, surrogacy is not regulated, apart from Brazil and Uruguay. In Brazil, surrogacy is allowed only in the altruistic regime. There are 2 circumstances in which a person can resort to surrogacy, a woman who has ovarian reproductive potential but a uterine condition that prevents pregnancy, or a same-sex couple. In either case, the surrogate must be a 1 ^st^ to 4 ^th^ degree relative of one of the PIs, such as mother, sister, aunt, or cousin. As a consequence of the lack of legislation banning commercial agreements, some countries, such as Colombia, have become popular surrogacy destinations in the last years ( Fig. 1 ). 206

### Europe

**Fig. 2:**
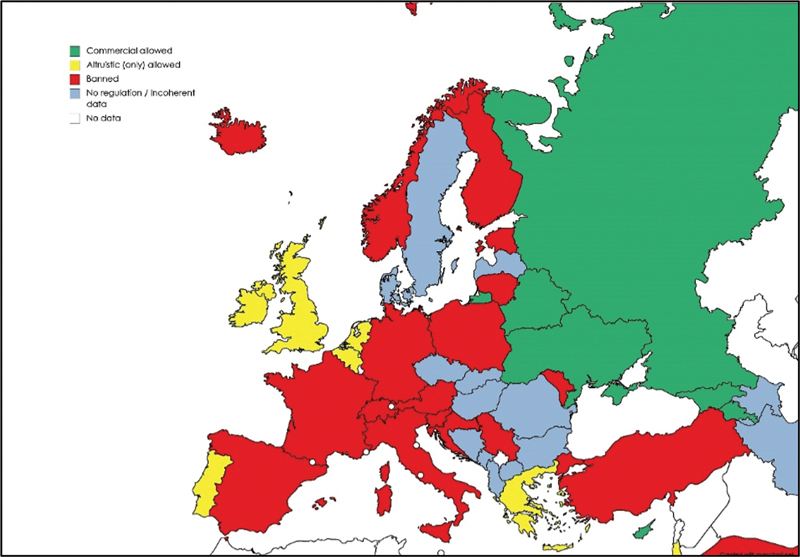
Map showing the current legal status of surrogacy in Europe.

Legislation in Europe varies between different countries. 207 Surrogacy in Europe is allowed or not banned in Albania, Armenia, Belarus, Belgium, Cyprus, Czech Republic, Georgia, Greece, Ireland, Macedonia, Portugal, Romania, Russia, the Netherlands, the UK, and Ukraine. 208 On the other hand, it is completely banned in Austria, Estonia, France, Germany, Italy, Lithuania, Norway, Spain, Sweden, and Switzerland. 157 In most countries where it is regulated, only altruistic surrogacy is permitted, such as Belgium, Greece, Ireland, the Netherlands, Portugal, and the UK ( Fig. 2 ). 170 208 209 In Georgia, Russia, and Ukraine commercial surrogacy is possible, but in general limited to heterosexual couples. 5 Georgia and Ukraine became a major destination for commercial surrogacy, due to its attractive prices and easiness of the process. 5 Since there is no uniform legislation, the European Court of Human Rights (ECtHR) has gained importance regarding transcontinental surrogacy for European citizens, especially for receiving countries with no specific legislation or where it is forbidden. 17 153 210 211 212 213 This entity has mediated some complicated processes, in particular in France, ultimately ruling in favor of the legal recognition of the nationality and affiliation of children conceived through international surrogacy, bearing in mind that this would be in their best interest. 85 143 214 215 216 In addition, in some countries such as the UK, courts have accepted foreign commercial surrogacy, as national legislation supports the concept of surrogacy, provided that the foreign surrogacy is lawful, there are adequate safeguards for the child, the interests of the child being paramount, the arrangements are ethical and not exploitative, and the costs are reasonable. 217 218 219 220 221 In 2009, Spain made an ad hoc regulation of the national registry to facilitate the often unpredictable process of recognition of the filiations resulting from cross-border surrogacy. 222 223 Norway does not allow surrogacy of any kind but recognizes the citizenship of children of Norwegian parents born by surrogacy abroad. 224

### Asia

**Fig. 3:**
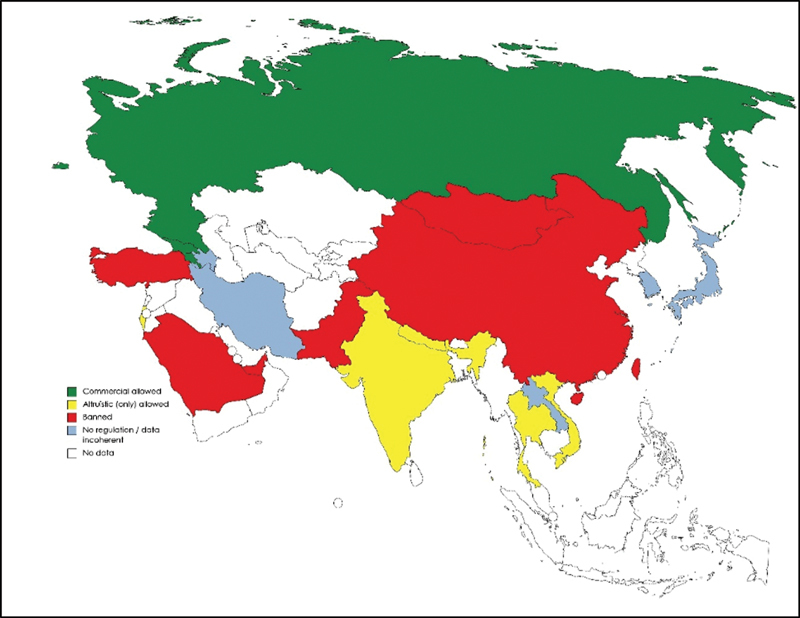
Map showing the current legal status of surrogacy in Asia.

Asian countries used to be a major destination for commercial surrogacy, until more restricted legislation on the subject has progressively been imposed. 225 226 Since 2018, when commercial and international surrogacy were both banned in India – the Surrogacy (Regulation) Bill – most countries in south or southeast Asia do not recognize commercial surrogacy. 227 228 229 230 231 India, Nepal, Thailand, and Vietnam recognize altruistic surrogacy (if not for all, in some specific situations or for national citizens only) but all these countries have explicitly banned commercial surrogacy. 173 232 233 Japan and South Korea do not have specific regulation regarding surrogacy. 8 234 Mongolia, Pakistan, People's Republic of China and Taiwan explicitly prohibit any kind of surrogacy. 235 Even though prohibited, in People's Republic of China there is an important practice of clandestine commercial surrogacy ( Fig. 3 ). 236 Nevertheless, an important part of these countries have no regulation at all regarding surrogacy, so it is not considered illegal and in some of them it keeps on being performed. 237 As a consequence of the ban to commercial surrogacy imposed by most south or southeast Asian countries, in particular India and Thailand, Laos became a popular choice, sometimes in an hybrid regimen with Thailand. 173 In the Middle East, Israel allows altruistic surrogacy only. 155 238 Iranian legislation is not clear regarding surrogacy and it is not an uncommon practice in the country. 179 In Saudi Arabia and in the United Arab Emirates, surrogacy is completely forbidden. 178 179

### Oceania

**Fig. 4:**
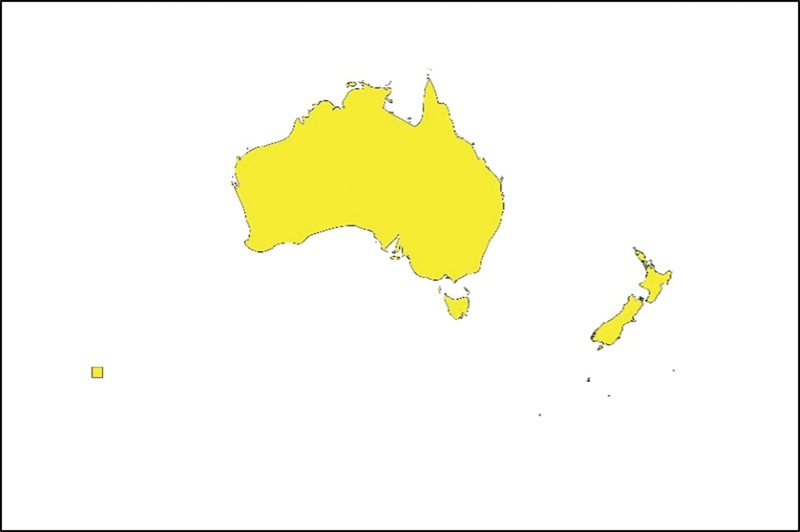
Map showing the current legal status of surrogacy in Oceania.

In Oceania, altruistic surrogacy may be performed in Australia and New Zealand, but commercial surrogacy is illegal ( Fig. 4 ). 232 239 240 241 242 243

### Africa

**Fig. 5:**
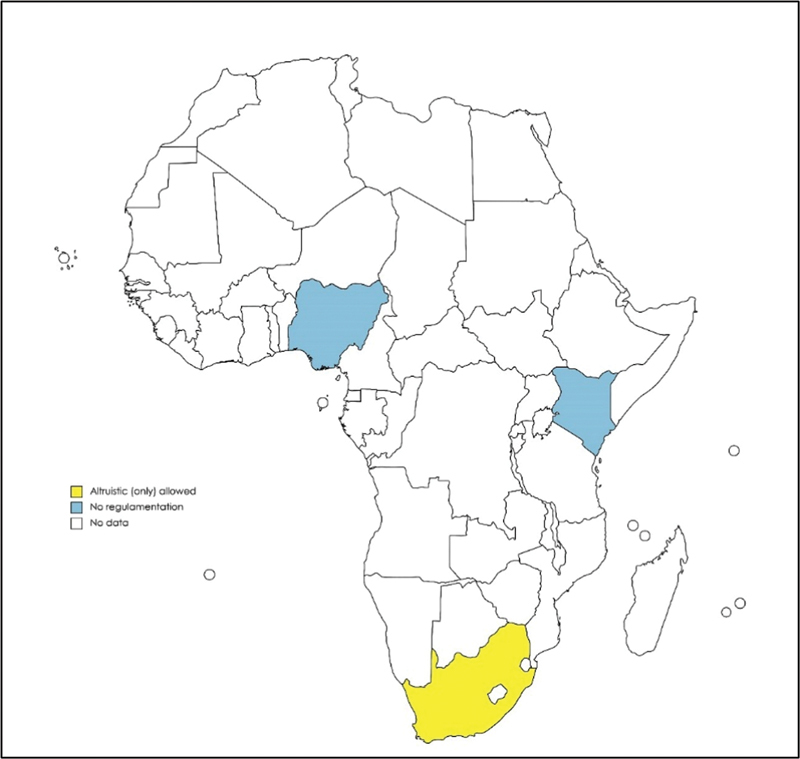
Map showing the current legal status of surrogacy in Africa.

Most African countries do not have any regulation concerning surrogacy. In Kenya, surrogacy is not regulated, hence it became a popular destination for this practice. 244 In South Africa, altruistic surrogacy is allowed ( Fig. 5 ). 157

## International Affairs

In order to avoid transnational surrogacy, some models of “ideal” commercial surrogacy laws and arrangements have been proposed. Some Australian groups proposed a model targeting a fair and just compensation, enforceability of surrogacy agreements, amended parentage presumptions and the ability to obtain prebirth parenting orders, regulation of surrogacy agencies and brokers, and recognition of approved international surrogacy arrangements. 245 246 Given the legal diversity and the frequent difficulty of fitting foreign activity into national law, there are many calls of action at the international, national, and professional levels to establish a human rights based system of international governance based on three regulatory models: public health monitoring, intercountry adoption, and trafficking in human beings, organs and tissues. 247 248 249 250 251 As stated before, many international intermediates make the connection between the IPs, the gametes donors and the carriers, most of the times via specific gamete banks, IVF clinics and surrogacy agencies. Most of these agencies are based in a unique country but they operate with IPs of any nationality, offering surrogacy programs in different countries, adjustable to any case.

## Conclusion

Surrogacy is an important means for some people to achieve biological parenthood, in particular women with uterine disorders, single men and male couples. However, this procedure entails important ethical dilemmas and legal issues. As a consequence of the diverse legal contexts worldwide, transnational surrogacy programs are frequently used, despite the possible legal complications. Commercial surrogacy is a common practice, although not regulated in most countries. This technique raises even more ethical and legal dilemmas. Various countries and international organizations made important attempts to regulate this practice in order to standardize its legal context worldwide and avoid litigation. Nevertheless, the situation remains largely unregulated and, as such, there is still a long way to go.
